# Origin of Symmetric Dimer Images of Si(001) Observed by Low-Temperature Scanning Tunneling Microscopy

**DOI:** 10.1038/srep27868

**Published:** 2016-06-13

**Authors:** Xiao-Yan Ren, Hyun-Jung Kim, Chun-Yao Niu, Yu Jia, Jun-Hyung Cho

**Affiliations:** 1 International Laboratory for Quantum Functional Materials of Henan, and School of Physics and Engineering, Zhengzhou University, Zhengzhou 450001, China; 2 Department of Physics and Research Institute for Natural Sciences, Hanyang University, 17 Haengdang-Dong, Seongdong-Ku, Seoul 133-791, Korea; 3 School of Mechanical and Electrical Engineering, Henan Institute of Science and Technology, Xinxiang 453003, China; 4 Korea Institute for Advanced Study, 85 Hoegiro, Dongdaemun-gu, Seoul 130-722, Korea; 5 Center for Advanced Analysis and Computational Science, Zhengzhou University, Zhengzhou 45001, China; 6 International Center for Quantum Design of Functional Materials (ICQD), HFNL, University of Science and Technology of China, Hefei, Anhui 230026, China

## Abstract

It has been a long-standing puzzle why buckled dimers of the Si(001) surface appeared symmetric below ~20 K in scanning tunneling microscopy (STM) experiments. Although such symmetric dimer images were concluded to be due to an artifact induced by STM measurements, its underlying mechanism is still veiled. Here, we demonstrate, based on a first-principles density-functional theory calculation, that the symmetric dimer images are originated from the flip-flop motion of buckled dimers, driven by quantum tunneling (QT). It is revealed that at low temperature the tunneling-induced surface charging with holes reduces the energy barrier for the flipping of buckled dimers, thereby giving rise to a sizable QT-driven frequency of the flip-flop motion. However, such a QT phenomenon becomes marginal in the tunneling-induced surface charging with electrons. Our findings provide an explanation for low-temperature STM data that exhibits apparent symmetric (buckled) dimer structure in the filled-state (empty-state) images.

Over the last 30 years the atomic and electronic structures of the Si(001) surface have been extensively investigated because of the fundamental building block for the fabrication of electronic devices as well as for the prototypical model system of semiconductor surfaces^1^,^2^,^3^,^4^,^5^,^6^,^7^,^8^. From enormous experimental and theoretical studies, it is well established that the basic reconstruction of Si(001) consists of the formation of buckled dimers^9^,^10^,^11^,^12^. However, at room temperature scanning tunneling microscopy (STM) experiments showed symmetric dimer images because of a thermally activated flip-flop motion of buckled dimers. Such apparent symmetric dimer images disappear below ~120 K^13^, forming either the *c*(4 × 2) structure [see Fig. 1(a)] consisting of alternatively buckled dimers along and perpendicular to the dimer rows or the *p*(2 × 2) one with alternatively buckled dimers along the dimer rows. Surprisingly, further cooling below ~20 K causes the buckled dimers to appear symmetric again^14^,^15^. Such symmetric-dimer STM images at low temperature have been explained in terms of various origins such as a dynamical flip-flop motion of buckled dimers^15^,^16^, local surface charging effects^17^, a possible asymmetric *p*(2 × 1) reconstruction^18^, an inelastic tunneling mechanism via electron-vibration coupling^19^, and a contribution of bulk states^20^,^21^. However, the microscopic mechanism underlying the low-temperature symmetric dimer images has remained an open question.

There have so far been a number of low-temperature STM experiments to characterize the apparent symmetric dimer images. Yokoyama and Takayanagi^15^ observed the symmetric dimer images at 5 K with both positive and negative bias voltages, which were explained by slow dynamical flip-flop motion of the buckled dimers during the STM scan. Mitsui and Takayanagi^16^ found that at 65 K higher tunneling currents increase not only the area of symmetric dimer images but also the flip-flop rate of buckled dimers regardless of the polarity of the bias voltage. However, Ono *et al*.^17^ observed both buckled and symmetric dimer images depending on the polarity of the bias voltage below 10 K: i.e., the buckled dimer images, locally forming *c*(4 × 2) or *p*(2 × 2) periodicity, were observed with positive bias voltages (empty-state images), while most of the dimers appear symmetric with negative bias voltages (filled-state images). Subsequent low-temperature STM experiments^22^,^23^,^24^ confirmed buckled dimer structure in the empty-state images and apparent symmetric dimer structure in the filled-state images. Interestingly, a recent STM study of Manzano *et al*.^21^ reported that at 7 K the negative bias voltages smaller than −1.5 V remained a *c*(4 × 2) reconstruction, but those larger than −1.5 V produced symmetric dimer images. On the basis of existing low-temperature STM data^15^,^16^,^17^,^21^, the following questions on the appearance of symmetric dimer images can be raised: i.e., Why does the activation barrier (*E*_*b*_) for the flipping of buckled dimers become much reduced at low temperature? What is the reason why the filled-state and empty-state STM images exhibit symmetric and buckled dimer structures, respectively? How does the tunneling-induced surface charging at low temperature^17^,^25^,^26^ or the electric field via bias voltage affect STM imaging to show apparent symmetric dimer structure?

In this paper, we perform first-principles density-functional theory (DFT) calculations to investigate the energy difference [equivalently *E*_*b*_ as shown in Fig. 1(b)] between the symmetric-dimer structure and the *c*(4 × 2) structure under electron or hole doping as well as in the presence of external electric field applied along the [001] direction. We find that, as the amount of hole doping increases, *E*_*b*_ decreases more dominantly than the case of electron doping. Compared to such surface charging effects, the application of electric field is found to give a relatively small change in *E*_*b*_. As *E*_*b*_ decreases with hole doping, the thermally activated flipping rate of buckled dimers is still negligible below 10 K, but the quantum tunneling (QT) driven flip-flop motion can be enabled to produce the symmetric-dimer STM images. Such a QT phenomenon of buckled dimers is, however, marginal with electron doping. Thus, a long-standing puzzle about the appearance of symmetric dimer images in low-temperature STM experiments can be solved in terms of the QT-driven flip-flop motion of buckled dimers, which can be facilitated by the tunneling-induced surface charging with holes.

## Results and Discussion

**Figure 1:**
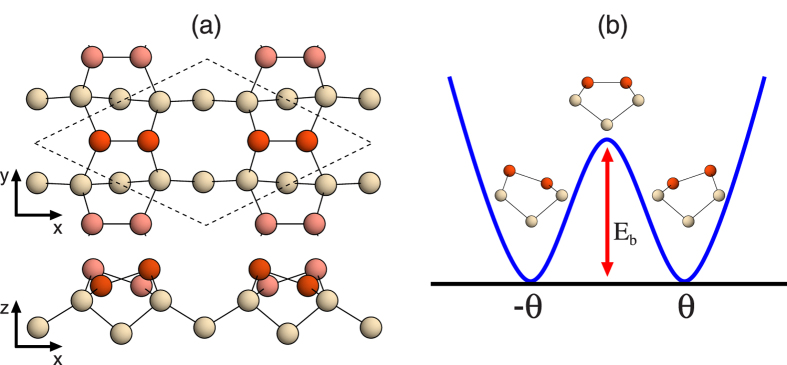
(**a**) Top and side views of the optimized *c*(4 × 2) structure of Si(001). The *c*(4 × 2) unit cell is indicated by the dashed line. The **x** (**y**) axis is perpendicular (parallel) to dimer rows, while the **z** axis is along the [001] direction. For distinction, the Si-dimer atoms within and outside the *c*(4 × 2) unit cell are drawn with two different dark circles. In (**b**), the symmetric double-well potential for the flipping of buckled dimers is schematically drawn. Here, *E*_*b*_ denotes the energy barrier, obtained by the energy difference between the *p*(2 × 1) and *c*(4 × 2) structures.

**Figure 2:**
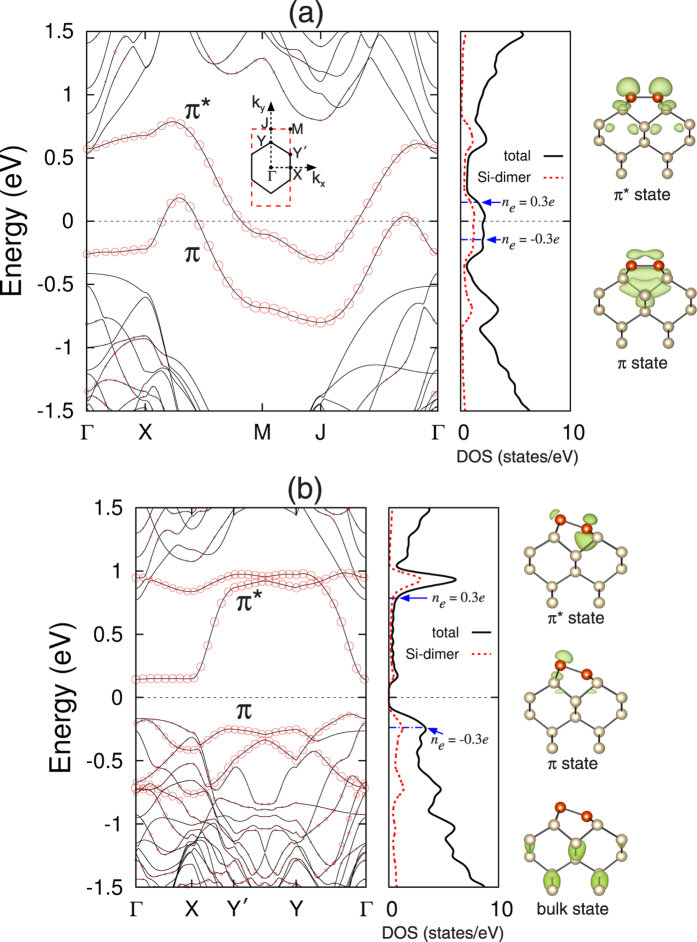
Calculated surface band structures of (**a**) the *p*(2 × 1) and (**b**) *c*(4 × 2) structures. The bands projected onto the *p*_*x*_, *p*_*y*_, and *p*_*z*_ orbitals of Si-dimer atoms are displayed with circles whose radii are proportional to the weights of such orbitals. The energy zero represents *E*_*F*_. The inset in (**a**) shows the surface Brillouin zones of the *p*(2 × 1) and *c*(4 × 2) unit cells. The total DOS and the local DOS of Si dimers are displayed with solid and dotted lines, respectively. The charge characters of the *π* and π* surface states at the Γ point are drawn with an isosurface of 0.05 *e*/Å, while that of the bulk state of *c*(4 × 2) at the Γ point (just below *E*_*F*_) is drawn with an isosurface of 0.02 *e*/Å.

We begin to optimize both the symmetric dimer structure, forming a *p*(2 × 1) periodicity (hereafter, designated as the *p*(2 × 1) structure), and the *c*(4 × 2) structure. The optimized *c*(4 × 2) structure is displayed in Fig. 1(a). We find that the *c*(4 × 2) structure has a dimer bond length of *d*_*D*_ = 2.357 Å and a dimer buckling angle of *θ* = 18.0°. This *c*(4 × 2) structure is found to be more stable than the symmetric-dimer structure by 255 meV per dimer, yielding *E*_*b*_ = 255 meV [see Fig. 1(b)]. As shown in Fig. 2(a,b), the calculated band structure of *p*(2 × 1) has a metallic band crossing the Fermi level *E*_*F*_, whereas that of *c*(4 × 2) exhibits a semiconducting feature with a band gap *E*_*g*_ of 0.27 eV. The present results for the geometry, energetics, and band structure of the *c*(4 × 2) structure are in good agreement with those of previous DFT calculations^10^,^27^.

**Figure 3:**
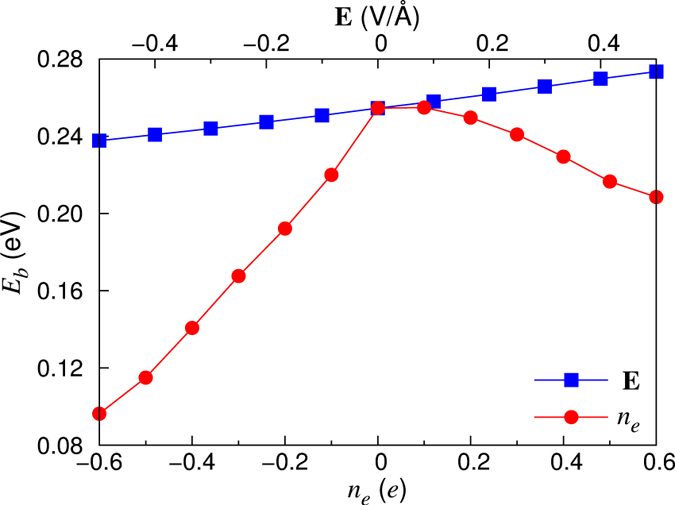
Calculated energy barrier *E*_*b*_ [seeFig. 1(b)] for the flip-flop motion of buckled dimers as a function of electron and hole dopings as well as external electric field. The unit of *e* in *n*_*e*_ is given per *p*(2 × 1) unit cell.

It has been known in several metal-adsorbate systems on Si or Ge surfaces^25^,^26^,^28^ that below ~40 K electrons or holes, injected through tunneling current in STM, result in surface charging due to a substantially suppressed charge transport between the surface layer and the semiconducting bulk. In order to examine the influence of surface charging on the energetics of the *p*(2 × 1) and *c*(4 × 2) structures, we perform total-energy calculations for the two structures with electron (whose amount is represented as a positive value of *n*_*e*_) or hole doping. Figure 3 shows the calculated values of *E*_*b*_ as a function of *n*_*e*_ ranging from −0.6*e* to 0.6*e* per *p*(2 × 1) unit cell. We find that both the electron and hole dopings reduce the energy difference between the *p*(2 × 1) and *c*(4 × 2) structures. The resulting decrease of *E*_*b*_ with electron or hole doping can be attributed to the metallic and semiconducting features of the *p*(2 × 1) and *c*(4 × 2) structures, respectively. As shown in Fig. 2(a,b), for the electron doping of *n*_*e*_ = 0.3*e*, excess electrons in *p*(2 × 1) occupy the electronic states just above the Fermi level, while those in the semiconducting *c*(4 × 2) structure occupy the conduction band separated by a band gap of 0.27 eV from the valence band. Therefore, the total energy of the *p*(2 × 1) structure is expected to decrease more largely compared to the *c*(4 × 2) structure. On the other hand, for the hole doping of *n*_*e*_ = −0.3*e*, holes in *p*(2 × 1) are created in the electronic states just below the Fermi level, while those in *c*(4 × 2) are created in the relatively lower valence bands. One thus expects a larger increase in the total energy of the *c*(4 × 2) structure compared to the *p*(2 × 1) structure. The resulting decrease of the energy difference between the *p*(2 × 1) and *c*(4 × 2) structures under either electron or hole dopings leads to a decrease of *E*_*b*_.

As shown in Fig. 3, *E*_*b*_ decreases more significantly with increasing hole doping, compared to the case of electron doping. This difference between electron and hole dopings may be explained in terms of the different characters of the unoccupied and occupied electronic states in the *p*(2 × 1) and *c*(4 × 2) structures: i.e., (*i*) the lowest unoccupied states in *p*(2 × 1) and *c*(4 × 2) are mostly the surface states of π* orbitals and (ii) the highest occupied states in *p*(2 × 1) are the surface states of *π* orbitals, while those in *c*(4 × 2) consist of the surface states of orbitals as well as the bulk states [see the total density of states (DOS) and the local DOS of Si dimers in Fig. 2(a,b)]. It is found that, for hole doping with *n*_*e*_ = −0.3*e*, the majority of the holes in the *c*(4 × 2) structure is created in the bulk states around the Γ point [see Fig. 1S of the Supplemental Material], possibly giving rise to a relatively larger strain energy compared to the *p*(2 × 1) structure where holes are created mostly in the surface states. Here, we note that, since the atom in the interior of the bulk has more neighboring atoms and experiences relatively larger interaction forces from its surroundings than the surface atom^29^, the hole-induced strain in *c*(4 × 2) is likely to yield a larger energy cost compared to the case of *p*(2 × 1). In this sense, we believe that hole doping decreases a more significant decrease of *E*_*b*_, compared to electron doping where excess electrons in both the *c*(4 × 2) and *p*(2 × 1) structures are occupied mostly in their surface states.

Next, we examine the influence of external electric field **E** on the energetics of the *p*(2 × 1) and *c*(4 × 2) structures. Here, **E** is simulated by superimposing an additional sawtooth potential along the [001] direction (taken as the +*z* direction) with discontinuity at the mid-plane of the vacuum region of the supercell. Note that an STM bias voltage of 1.5 V and a tip-sample distance of ~5 Å would give rise to an electrical field of ~0.3 V/Å. Figure 3 also shows the calculated values of *E*_*b*_ as a function of **E** ranging between −0.5 and +0.5 V/Å. We find that *E*_*b*_ increases (decreases) as **E** increases along the +*z* (−*z*) direction. These different behaviors of *E*_*b*_ depending on the direction of **E** can be explained in terms of the different contributions of electrostatic energy due to external electric field between the *p*(2 × 1) and *c*(4 × 2) structures. Since the surface dipole moment p_*z*_ (pointing −*z* direction) of the metallic *p*(2 × 1) structure is larger in magnitude by Δp_*z*_ = 0.038 *e*Å than that of the semiconducting *c*(4 × 2) structure, an electric field applied along the +*z* (−*z*) direction gives a positively (negatively) larger electrostatic energy *U* = −**p** · **E** of surface dipole in *p*(2 × 1) compared to in *c*(4 × 2), leading to an increase (decrease) of *E*_*b*_. Here, we evaluate the difference of surface dipole moment Δp_*z*_ between the *p*(2 × 1) and *c*(4 × 2) structures by using the relation with the corresponding work function change ΔW^30^: i.e. 
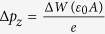
, where A is the surface area of *p*(2 × 1) unit cell and ΔW between the *p*(2 × 1) and *c*(4 × 2) structures is 0.456 eV. We find that the variation of *E*_*b*_ with respect to the external electric field of 0 ≤ |**E**| ≤ 0.5 V/Å is less than ~20 meV, much smaller than that (~160 meV) obtained from hole doping (see Fig. 3). Thus, we can say that the influence of hole doping on *E*_*b*_ is much more pronounced than that arising from external electric field.

It should be noted that, in order to explain their observed symmetric-dimer STM images, Yokoyama and Takayanagi^15^ suggested that anharmonic potential effects would reduce the barrier height to induce the dynamical dimer flipping. However, this mechanism cannot explain the STM observations of buckled and symmetric dimer images depending on the polarity of the bias voltage^17^,^21^,^22^,^23^,^24^. Moreover, according to the DFT calculation of Bokes *et al*.^31^, the energy difference Δ*E* between the symmetric and buckled dimer structures varies by only ~0.01 eV/dimer with changing the lattice parameter of ±1%. Since this value of Δ*E* is much smaller than those obtained in the present case of charge doping, the energy change associated with the negative thermal expansion is unlikely to explain the observed symmetric-dimer STM images.

To account for the symmetric dimer images observed from low-temperature STM experiments^15^,^16^,^17^,^21^, we investigate the flip-flop motion of buckled dimers driven by either thermal activation^32^ or quantum tunneling. For this, we employ a symmetric double-well potential [see Fig. 1(b)] that describes the potential energy surface of flipping dimers as a function of *θ*. This potential surface is confirmed by the nudged elastic band calculations^33^,^34^ for *n*_*e*_ = 0, 0.3*e*, and −0.3*e*, where the *p*(2 × 1) structure is in unstable equilibrium, showing that there is no energy barrier between the *p*(2 × 1) and *c*(4 × 2) structures. Using a harmonic approximation, we obtain a vibration frequency for this potential well as 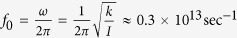 in the absence of electron or hole doping, where the torsion constant *k* and the inertia moment *I* of flipping dimer can be estimated from 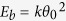 (*θ*_0_: dimer buckling angle at the lowest-energy configuration) and 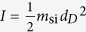 (*m*_si_: mass of Si atom). Based on an Arrhenius-type activation process, a thermally excited flipping rate can be expressed as 
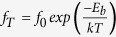
. With the calculated values of *E*_*b*_ and *f*_0_ as a function of |*n*_*e*_| ≤ 0.6*e*, we obtain *f*_*T*_ smaller than 0.8 × 10^−36^ sec^−1^ at 10 K. This thermal flipping rate is too small to explain the observed symmetric-dimer STM images with flicker noise^15^,^16^. As an alternative explanation for the flip-flop motion of buckled dimers, we consider quantum tunneling (QT) within the double-well potential, whose flipping rate can be approximated^35^,^36^ as


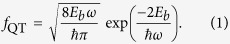


**Figure 4:**
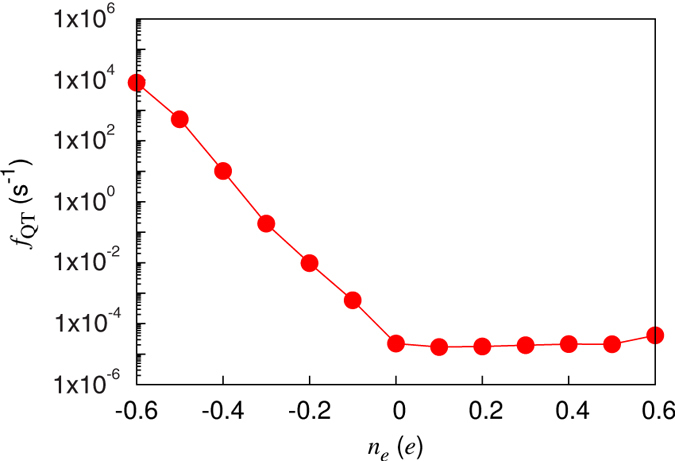
Calculated QT-driven flipping rate of buckled dimers as a function of electron and hole dopings.

**Figure 5:**
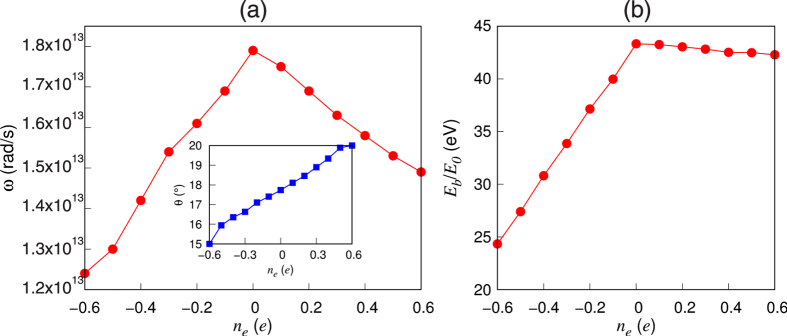
(**a**) Zero-point frequency *ω* and (**b**) the ratio of the energy barrier *E*_*b*_ and the zero-point energy *E*_0_ 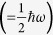. The inset in (**a**) shows the dimer buckling angle as a function of electron and hole dopings.

Contrasting with *f*_*T*_, *f*_QT_ is independent of temperature, while it is determined by the ratio of *E*_*b*_ and the zero-point energy *E*_0_ 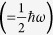. In Fig. 4, the estimated values of *f*_QT_ are plotted as a function of *n*_*e*_. We find that *f*_QT_ sharply increases with increasing hole doping, while it is nearly flat with respect to electron doping. Here, we note that *ω* obtained from the calculated values of *E*_*b*_ and *θ*_0_ [see Fig. 5(a)] gives rise to a large decrease in the ratio of *E*_*b*_ and *E*_0_ under hole doping, but hardly changes *E*_*b*_/*E*_0_ under electron doping [see Fig. 5(b)]. Since hole doping with *n*_*e*_ = −0.6*e* gives a relatively smaller ratio of *E*_*b*_/*E*_0_ ~ 23 compared to that (~42) over the entire range of electron doping, *f*_QT_ significantly increases by eight orders of magnitude upon hole doping (see Fig. 4). For hole doping with |*n*_*e*_| > 0.5*e*, *f*_QT_ becomes greater than ~5.1 × 10^2^ sec^−1^. Considering that it takes *τ*_dimer_ ≈ 10^−2^ sec to obtain an STM image of a dimer^37^,^38^, such a hole-doping induced flip-flop motion can produce the observed symmetric dimer images in low-temperature STM experiments^15^,^16^,^17^,^21^. It is noted that, as temperature increases above ~40 K, surface charging effects begin to disappear, therefore giving rise to *f*_QT_ ~2.2 × 10^−5^ sec^−1^ computed at *n*_*e*_ = 0 (see Fig. 4). This indicates that the QT-driven flipping motion becomes weakened above ~40 K, leading to the appearance of buckled-dimer STM images. In particular, Manzano *et al*.’s observation^21^ of buckled dimer images at voltages lower than −1.5 V can be explained by the fact that the hole doping induced by small negative bias voltages can reduce the energy barrier *E*_*b*_, but not so much so that the flip-flop motion is enabled to produce symmetric dimer images. Meanwhile, according to the low-temperature STM experiment of Yoshida *et al*.^39^, the flip-flop frequency depends both on gap voltage and tunneling current. Here, we note that at positive bias voltages the dynamic behavior of the dimers was observable. Interestingly, such effects of gap voltage and tunneling current in STM measurement was observed in a different surface system Sn/Ge(111)^40^. It is noticeable that, according to Yoshida *et al*.’s STM experiment^39^, the flip-flop frequency as a function of the positive-bias voltage and tunneling current (between 0.2 nA and 1 nA) is relatively much smaller than that as a function of the negative-bias voltage. This difference of the flip-flop frequency with respect to the bias-polarity is not only qualitatively consistent with the present simulation results, but it may also explain why some STM experiments^17^,^21^,^22^ easily observed the symmetric dimer images with negative-bias voltages. Also, the relatively smaller flip-flop frequency as a function of tunneling current may provide an explanation for why Ono *et al*.’s STM experiment^17^ observed no change of images with respect to tunneling current below 0.3 nA.

We note that the application of **E** along the −*z* (+*z*) direction decreases (increases) *E*_*b*_. Consequently, one expects that negative sample bias (equivalently, negative electric field) inducing hole doping at low temperature enhances the magnitude of *f*_QT_. On the other hand, positive sample bias (positive electric field) inducing electron doping suppresses *f*_QT_. These drastically different aspects of negative and positive bias voltages in low-temperature STM experiments account for the observations of symmetric and buckled dimer images in filled-state and empty-state images, respectively^17^,^21^.

Although we present a simple picture of the QT-driven flip-flop motion of buckled dimers with a double-well potential, we believe that it captures the microscopic mechanism underlying low-temperature symmetric-dimer STM images, as explained above. It is noted that the present DFT-GGA calculation may tend to somewhat overestimate the energy gain due to buckling. Indeed, the quantum Monte Carlo calculation^41^ which accurately describes electronic correlations extrapolates the value of *E*_*b*_ up to ~150 meV per dimer, in good agreement with the experimental estimates^32^. This reduction of *E*_*b*_ can enhance the QT-driven flip-flop motion of buckled dimers.

## Conclusions

In conclusion, we have performed a DFT-GGA calculation for the Si(001) surface to investigate the energy difference between the symmetric-dimer structure and the *c*(4 × 2) structure under electron or hole doping as well as applied external electric field along the [001] direction. This energy difference corresponding to the energy barrier for the flipping of buckled dimers was found to decrease more significantly with respect to hole doping compared to electron doping. Consequently, we found that hole doping gives rise to a sizable QT-driven frequency of the flip-flop motion of buckled dimers while electron doping shows the marginal QT effects. These different QT aspects of hole and electron dopings are most likely to yield the imaging difference between the filled- and empty-state STM images at low temperature. Thus, we concluded that quantum tunneling enhanced by the tunneling-induced hole doping causes the observation of symmetric dimer images in low-temperature STM experiments, but, as temperature increases, such a surface charging effect becomes weakened, thereby leading to an appearance of asymmetric dimer images.

## Methods

The present DFT calculations were performed using the FHI-aims code^42^ for an accurate, all-electron description based on numeric atom-centered orbitals, with “tight” computational settings. For the exchange-correlation energy, we employed the generalized gradient approximation of Perdew-Burke-Ernzerhof (PBE)^43^. The Si(001) surafce (with the Si lattice constant *a*_0_ = 5.418 Å) was modeled by a twelve-layer slab with ~30 Å of vacuum in between the slabs, where each Si atom in the bottom layer was passivated by two H atoms. The *k*-space integrations for the *p*(2 × 1) and *c*(4 × 2) structures were done equivalently with 32 k points in the surface Brillouin zone of the *p*(2 × 1) unit cell. Here, for the total-energy calculation of the *c*(4 × 2) structure, we employed the equivalent *p*(4 × 2) unit cell whose surface area is twice as large as that of the *c*(4 × 2) structure. We used a dipole correction that cancels the artificial electric field across the slab. All atoms except the bottom two layers were allowed to relax along the calculated forces until all the residual force components were less than 0.02 eV/Å. For the simulation of surface charging, we used the virtual crystal approximation^44^ to compensate excess electrons *n*_*e*_ or holes, where the nuclear charge of Si atoms is modified by a small amount Δ*Z* = *n*_*e*_/*N* (*N*: number of Si atoms within four deeper atomic layers). We note that all the simulations with hole or electron doping have been performed without spin-polarization. In order to check the possibility of spin-polarization, we performed the spin-polarized simulations for the doping concentrations of |*n*_*e*_| ≤ 0.6*e*. We found that the ground structure is nonmagnetic in the range of |*n*_*e*_| ≤ 0.5*e*, but it becomes spin-polarized at |*n*_*e*_| = 0.6*e* with a small magnetic moment of ~0.1 *μ*B. However, in the latter spin-polarized cases, the energy barrier between the *p*(2 × 1) and *c*(4 × 2) structures changes little by less than ~1 meV compared to the corresponding nonmagnetic cases.

## Additional Information

**How to cite this article**: Ren, X.-Y. *et al*. Origin of Symmetric Dimer Images of Si(001) Observed by Low-Temperature Scanning Tunneling Microscopy. *Sci. Rep.* **6**, 27868; doi: 10.1038/srep27868 (2016).

## Supplementary Material

Supplementary Information
