# Supplementary material for: Origin of Symmetric Dimer Images of Si(001) Observed by Low-Temperature Scanning Tunneling Microscopy
Source: Sci Rep. 2016 Jun 13;6:27868. doi: 10.1038/srep27868 (PMC4904415; doi:10.1038/srep27868)
Supplement: Supplementary Information [file srep27868-s1.pdf]

Supplemental Material for "Origin of Symmetric Dimer Images of Si(001) Observed by Low-Temperature Scanning Tunneling Microscopy"

Xiao-Yan Ren,[1,2,3] Hyun-Jung Kim,[2,4] Chun-Yao Niu,[1,5] Yu Jia*,[1,5] and Jun-Hyung Cho*[2,1,6]

[1] *International Laboratory for Quantum Functional Materials of Henan, and School of Physics and Engineering, Zhengzhou University, Zhengzhou 450001, China*

[2] *Department of Physics and Research Institute for Natural Sciences, Hanyang University, 17 Haengdang-Dong, Seongdong-Ku, Seoul 133-791, Korea*

[3] *School of Mechanical and Electrical Engineering, Henan Institute of Science and Technology, Xinxiang 453003, China*

[4] *Korea Institute for Advanced Study, 85 Hoegiro, Dongdaemun-gu, Seoul 130-722, Korea*

[5] *Center for Advanced Analysis and Computational Science, Zhengzhou University, Zhengzhou 45001, China*

[6] *International Center for Quantum Design of Functional Materials (ICQD), HFNL, University of Science and Technology of China, Hefei, Anhui 230026, China*

(Dated: May 3, 2016)

1. Band structures of the $c(4\times2)$ structure with a hole doping of $n_e = -0.3e$.

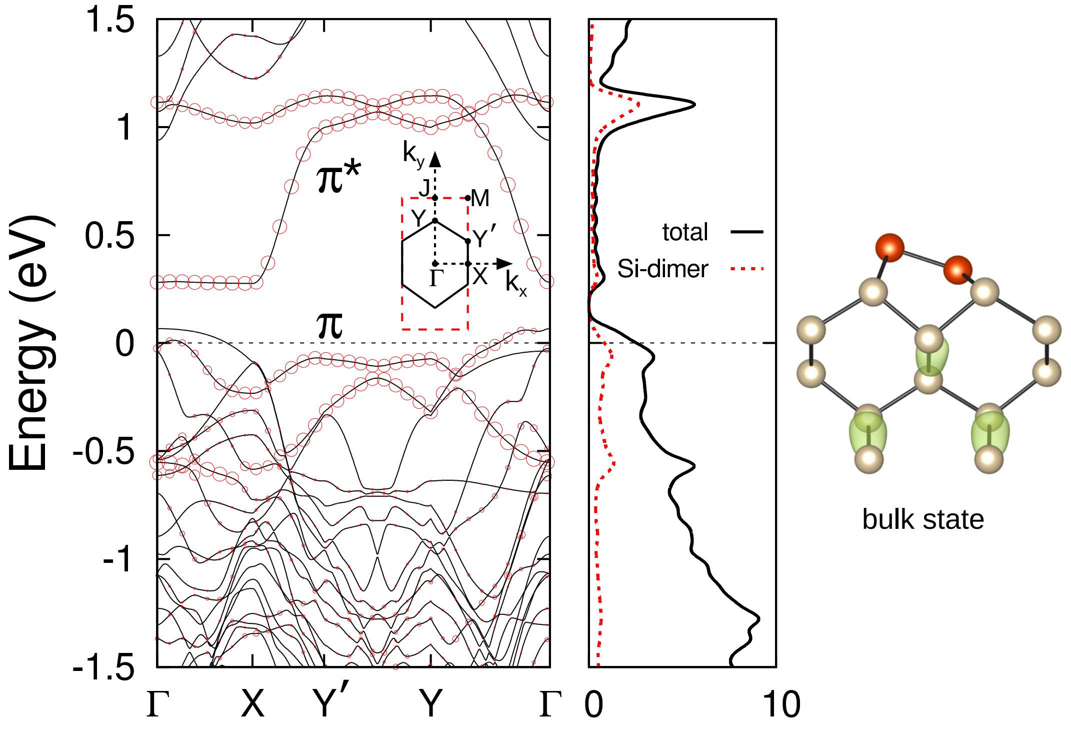

FIG. 1S: (Color online) Calculated surface band structure of the $c(4\times2)$ structure with a hole doping of $n_e = -0.3e$ per $p(2\times1)$ unit cell. The bands projected onto the p_x, p_y, and p_z orbitals of Si-dimer atoms are displayed with circles whose radii are proportional to the weights of such orbitals. The energy zero represents the Fermi level. The inset in (a) shows the surface Brillouin zones of the $c(4\times2)$ unit cell. The total DOS and the local DOS of Si dimers are displayed with solid and dotted lines, respectively. The charge character of the bulk state at the Γ point (just above E_F) is drawn with an isosurface of 0.02 e/Å.
